# Fully Integrated Biopotential Acquisition Analog Front-End IC

**DOI:** 10.3390/s151025139

**Published:** 2015-09-30

**Authors:** Haryong Song, Yunjong Park, Hyungseup Kim, Hyoungho Ko

**Affiliations:** Department of Electronics, Chungnam National University, Daejeon, 305-764, Korea; E-Mails: zealshr@cnu.ac.kr (H.S.); pyjj90@cnu.ac.kr (Y.P.); hanafos24@cnu.ac.kr (H.K.)

**Keywords:** biopotential, capacitively-coupled chopper instrumentation amplifier (CCIA), DC servo loop (DSL), ripple reduction loop (RRL), capacitive input boosting loop (CIBL)

## Abstract

A biopotential acquisition analog front-end (AFE) integrated circuit (IC) is presented. The biopotential AFE includes a capacitively coupled chopper instrumentation amplifier (CCIA) to achieve low input referred noise (IRN) and to block unwanted DC potential signals. A DC servo loop (DSL) is designed to minimize the offset voltage in the chopper amplifier and low frequency respiration artifacts. An AC coupled ripple rejection loop (RRL) is employed to reduce ripple due to chopper stabilization. A capacitive impedance boosting loop (CIBL) is designed to enhance the input impedance and common mode rejection ratio (CMRR) without additional power consumption, even under an external electrode mismatch. The AFE IC consists of two-stage CCIA that include three compensation loops (DSL, RRL, and CIBL) at each CCIA stage. The biopotential AFE is fabricated using a 0.18 µm one polysilicon and six metal layers (1P6M) complementary metal oxide semiconductor (CMOS) process. The core chip size of the AFE without input/output (I/O) pads is 10.5 mm^2^. A fourth-order band-pass filter (BPF) with a pass-band in the band-width from 1 Hz to 100 Hz was integrated to attenuate unwanted signal and noise. The overall gain and band-width are reconfigurable by using programmable capacitors. The IRN is measured to be 0.94 µV_RMS_ in the pass band. The maximum amplifying gain of the pass-band was measured as 71.9 dB. The CIBL enhances the CMRR from 57.9 dB to 67 dB at 60 Hz under electrode mismatch conditions.

## 1. Introduction

Recently, human-computer interface applications have gained significant attention. Many major global companies have developed various individualized bio-signal measuring applications. Biopotential detection devices enable continuous monitoring of various physiological information from a user; therefore, the biopotential detection circuits can be utilized in fields like medical, entertainment, and sports fields [1,2]. In addition, with integrated circuit (IC) process development, researches in highly miniaturized and low power consumption biopotential detection circuits have rapidly grown.

**Figure 1:**
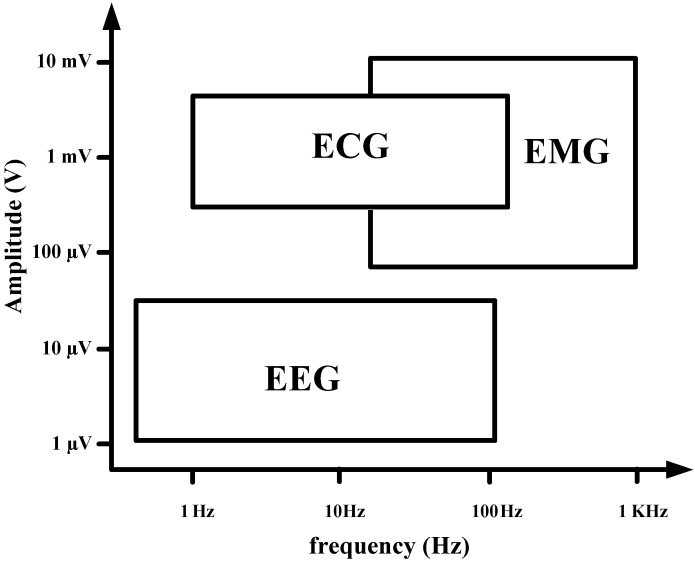
Amplitude and frequency band of bio-signal.

Most biopotential circuits commonly suffer from degraded performance because of the flicker noise in the bio-signal band, offset due to process variation, skin-to-electrode offset, and motion artifact signals from different body and cable motions during signal recording. In particular, biopotentials such as electroencephalogram (EEG), electrocardiogram (ECG), and electromyogram (EMG) have low-frequency (< 300 Hz) features that include flicker noise band, as shown in Figure 1.

DC components are not included in most biopotential signals; thus, the biopotential detection circuit generally adopts an AC-coupled input stage. The AC-coupled input stage with first-order high-pass characteristics can be simply implemented using capacitor pairs.

To effectively attenuate the unwanted components out of the biopotential signal band, a higher order high-pass filter (HPF) with a sub-Hz cut-off frequency is required. The HPF can be realized using very large resistors of a few GΩ using MOS-bipolar devices [3]. The sub-Hz low-frequency artifact signals such as respiration are simultaneously recorded with the bio-signal, and degrade the signal quality. A higher-order HPF is one of the effective solutions to attenuate these low frequency artifact signals. In addition, many bio-signal detection circuits were reported to overcome external offsets caused by polarization of the skin-electrode interface, and internal offsets caused by process variations [4,5,6]. For a comfortable connection between circuit and body, dry electrode rather than wet electrode is used in recent biopotential measuring device. The dry electrodes, however, have much higher output impedance than wet electrodes; thus an input stage with a much higher input impedance (larger than a few GΩ) is required for a high-quality biopotential acquisition. 

**Figure 2:**
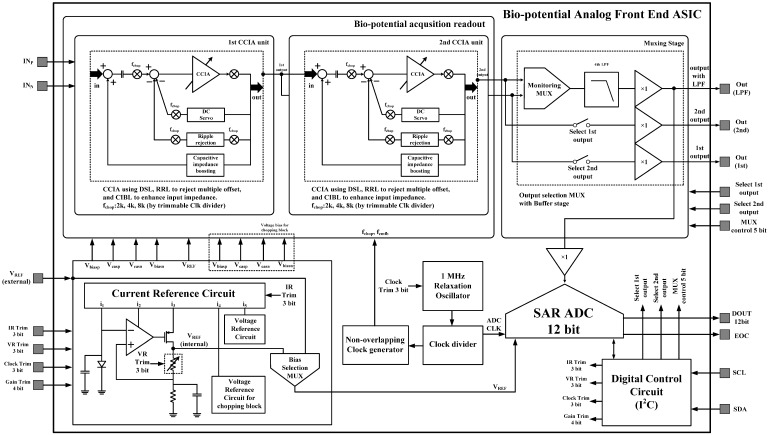
Architecture of a simplified biopotential acquisition circuit.

The overall architecture of the proposed AFE IC is shown in Figure 2. The proposed AFE adopts a multiple-offset compensation circuit to reduce both the internal and external offsets. The AFE consists of a two-stage capacitively coupled instrumentation amplifier (CCIA) with multiple offset compensation circuits at each stage. The default gain of a single CCIA is 36 dB. The single CCIA has a first-order HPF and DC servo loop (DSL), which generates additional poles; thus, a second-order HPF can be implemented in each CCIA stage. Previous studies on DSL required external capacitors [6]. In this design, DSL is fully integrated without external components using a differential Miller integrator with pseudoresistors and frequency shifting choppers. Chopper stabilization (CHS) is adopted for achieving sub-µV input referred noise (IRN). The operation of CHS in the CCIA, however, can generate a ripple voltage due to the offset, which is generated by a component mismatch. The mismatch can be suppressed by adding a ripple rejection loop (RRL). Previous RRLs were implemented using large coupling capacitors [5]. In this design, the RRL is implemented using a differential Miller integrator with pseudoresistors and frequency shifting choppers; thus, the circuit area is reduced because the large coupling capacitors can be eliminated. Insufficient input impedance is enhanced by a capacitive impedance boosting loop (CIBL). The previously reported active impedance boosting sub-circuit using positive feedback requires additional power consumption [4]. The CIBL in this design is implemented using a passive capacitor, and does not require additional power consumption. The CIBL also increases the CMRR, even under input electrode mismatch conditions. The IRN is minimized by an iterative noise optimization design procedure. Out-of-band biosignals are attenuated by a fourth-order BPF. This BPF consists of a fourth-order high-pass filter (HPF) followed by a cascaded two-stage CCIA, and a fourth-order Sallen-key low-pass filter (LPF) followed by a two-stage CCIA. The presented AFE is designed to be fully integrated and is fabricated using a standard 0.18-µm complementary metal oxide semiconductor (CMOS) process.

## 2. Circuit Design

### 2.1. Capacitively Coupled Chopper Instrumentation Amplifier (CCIA)

**Figure 3:**
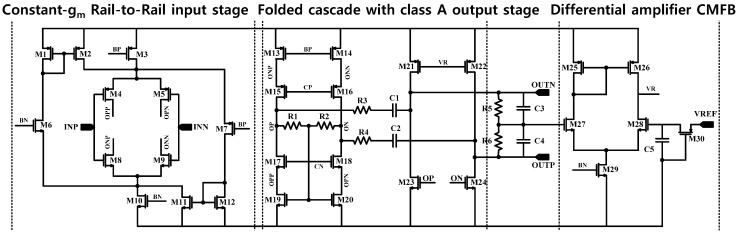
Schematic of proposed core amplifier.

A schematic of the core operational amplifier used in the CCIA is shown in Figure 3. The constant-transconductance (g_m_), rail-to-rail, folded-cascode with a class-A output stage is adopted for the amplifier. The core amplifier exploits the constant-gm rail-to-rail input stage to maximize the input swing range. The class-A output stage, followed by the folded cascode stage, provides a wider output swing range. The strong common mode feedback (CMFB) circuit is required to hold the output common mode to the reference voltage (VREF), assuming that the common mode fluctuates during the activation of chopper stabilization. The proposed amplifier adopts a dual CMFB structure: The first CMFB consists of a resistive CMFB of R1 and R2 at the cascode output stages, and the second CMFB consists of a differential amplifier CMFB at the class-A output stage.

To minimize the non-linearity of the input common mode voltage, the constant-gm rail-to-rail input stage is designed using 12 transistors (M1 through M12). From the supply voltage to ground voltage, the input stage supplies a constant bias current by the constant-g_m_ rail-to-rail circuit. The folded cascode output stage consists of eight transistors (M13 through M20) and two resistors (R1 and R2), which configure the resistive CMFB. The four transistors (M21 through M24) comprise the class-A output stage. Two resistors (R3 and R4) and two capacitors (C1 and C2) were added for frequency compensation. Five transistors (M25 through M29), two resistors (R5 and R6), and two capacitors (C3 and C4) constitute the additional CMFB. The open loop gain and the phase margin of the core amplifier are 70 dB and 70°, respectively.

**Figure 4:**
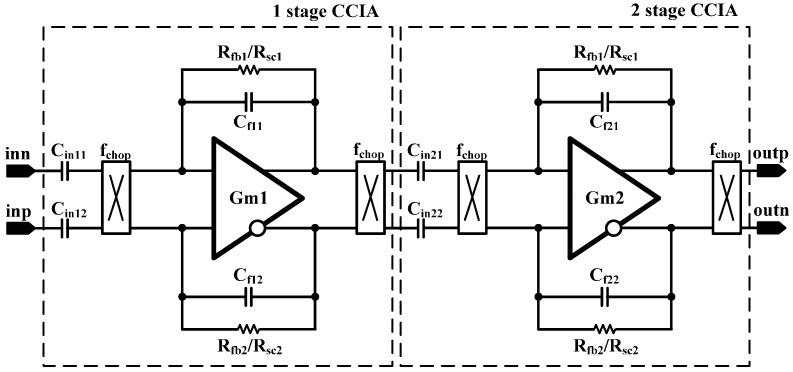
Schematic of the two-stage capacitively coupled chopper instrumentation amplifier (CCIA).

A simplified block diagram of the two-stage CCIA is shown in Figure 4. The CCIA consists of a fully differential amplifier, input capacitors, feedback capacitors, and pseudoresistors. The gain can be adjusted by programming the binary weighted feedback capacitor. Both the selectable pseudoresistor and switched capacitor (SC) feedback resistors are added to realize a large resistance value in the small integration area. The feedback resistors determine the cutoff frequency of the high-pass filter in the CCIA. The overall gain of the two-stage cascaded CCIA is 71.9 dB.

**Figure 5:**
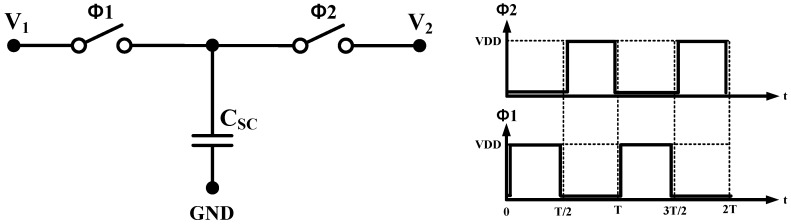
Schematic and timing diagram of the SC resistor circuit.

A switched capacitor resistor scheme and a pseudoresistor scheme can be used to implement a large-value resistor of a few gigaohms. Figure 5 shows the schematic of the SC resistor. The advantage of the SC resistor is high accuracy of time constants, voltage linearity, and temperature characteristics. In the SC resistor scheme, however, the noise level is higher than in the pseudoresistor scheme due to the high-frequency noise folding, which is a major disadvantage. Although the pseudoresistor can achieve a low noise level, the resistance of the pseudoresistor is significantly affected by process variations. In this design, the SC resistor scheme is exploited to obtain robustness to process variation and to obtain high accuracy. The SC resistor is realized by a minimum capacitor size of 16 femtofarads. The dummy capacitors are surrounded by the main capacitor to minimize process variation.

**Figure 6:**
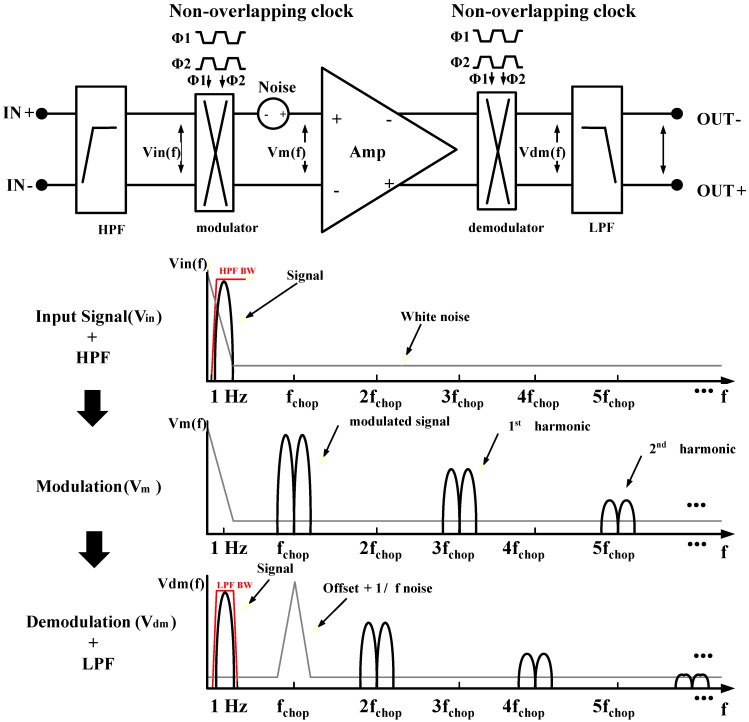
Principle of chopper stabilization.

**Figure 7:**
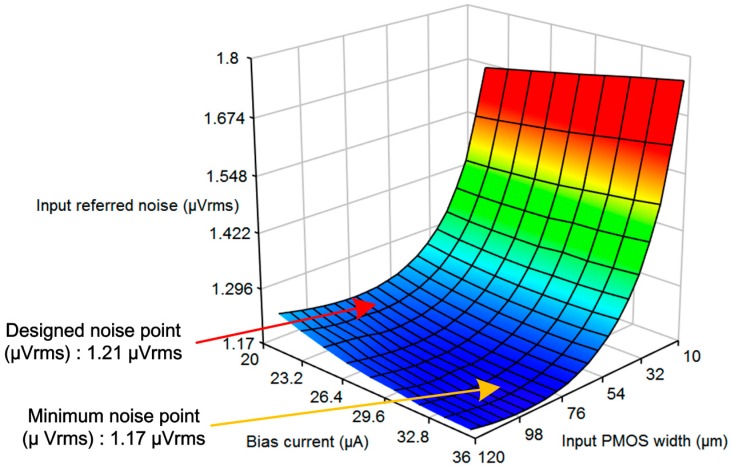
IRN optimization with input PMOS width and bias current.

Noise reduction is a key issue for achieving high-quality bio-potential acquisition. The core amplifier is designed to minimize flicker noise by sizing the transistors and varying the bias current. The modulated noise, the spikes, and the ripples after demodulation are eliminated by the LPF. Modulation and demodulation choppers are added to achieve low IRN in the amplification stage. To reduce the IRN, the CHS technique is adopted, as shown in Figure 6. The input signal is modulated to the high-frequency band, whereas the-low frequency noise components are still in the baseband. The modulated input signal is amplified and demodulated to the baseband. At the demodulation stage, the low-frequency flicker noise is modulated to the high-frequency band. The IRN is optimized using the two variables, input p-type metal oxide semiconductor (PMOS) width and the bias current of the input stage, as shown in Figure 7. The final value of the input PMOS width and the bias current are selected to be 60 μm and 20 μA, respectively. 

### 2.2. Bio-Signal Optimized Compensation Circuit

#### 2.2.1. DC Servo Loop (DSL) 

Biopotential signals suffer from high common-mode interference and the differential electrode offset coming from the differential body potentials at each electrode. Most biopotential amplifiers have high gain, and thus a small DC offset at the input stage often leads to output saturation. The DSL is designed to remove the external offset. 

**Figure 8:**
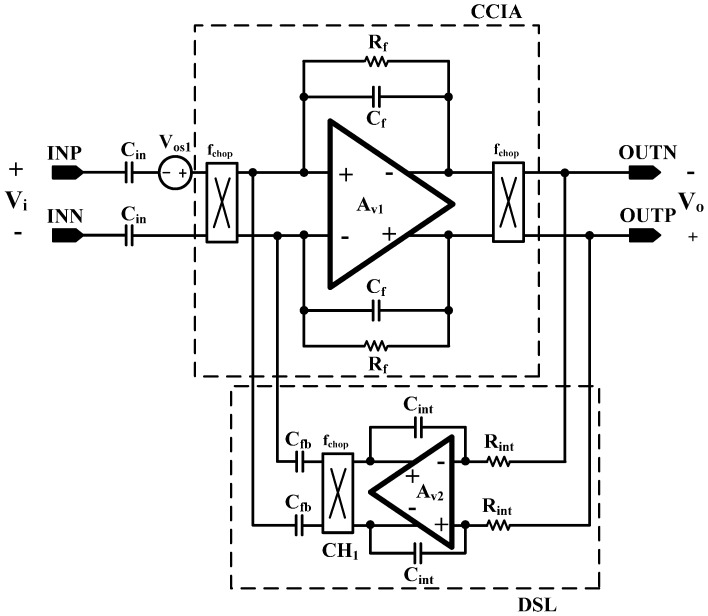
Schematic of the DSL.

The schematic of the DSL is shown in Figure 8. The operation of the DSL is as follows. The input offset voltage, Vos1, is amplified by the CCIA. The amplified offset is integrated by the fully differential Miller integrator. The integrated offset is modulated to the high frequency by CH1 and negatively fed back to the input stage. The transfer function without DSL and the transfer function with DSL can be expressed as Equations (1) and (2), respectively:

(1) $$\frac{v_{o}(s)}{v_{i}(s)}=-\frac{sR_{f}C_{i}}{1+sR_{f}C_{f}}$$

(2) $$\frac{v_{o}(s)}{V_{i}(s)}=\frac{-S^{2}\cdot R_{f}\cdot C_{fb}\cdot R_{\mathrm{int}}\cdot C_{\mathrm{int}}\cdot C_{i}}{\left(1+sR_{f}C_{f})\cdot (s\cdot R_{\mathrm{int}}\cdot C_{\mathrm{int}}\cdot C_{fb}-C_{f}\right)}$$

**Figure 9:**
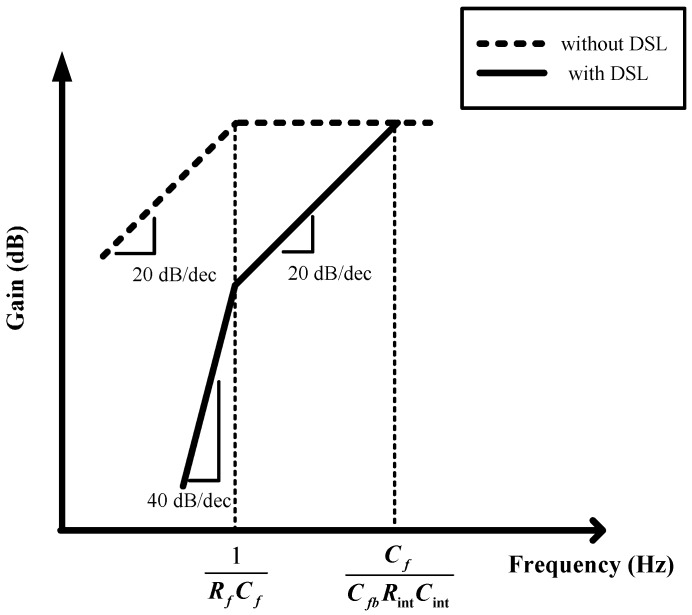
Frequency response of the CCIA.

The additional pole on the frequency of C_f_/(C_fb_∙R_int_∙C_int_) is generated by the DSL, as shown Figure 9, thus the second-order HPF is implemented. Low-frequency artifacts, such as respirations, are effectively attenuated by this second-order HPF. Therefore, second-order per each CCIA stage can be implemented using DSL and an AC-coupled input stage.

#### 2.2.2. Ripple Rejection Loop (RRL)

**Figure 10:**
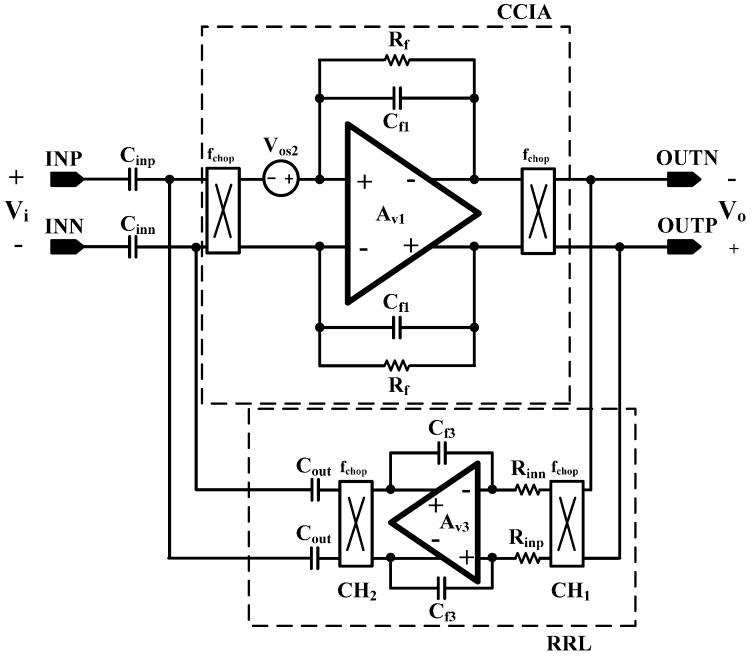
Schematic of the RRL.

The input transistor mismatch in CCIA causes the offset and becomes a “ripple” at the output stage by the demodulation chopper. The schematic of the RRL is shown in Figure 10. The chopper induced offset, “ripple”, is demodulated to the baseband by the input chopper of the RRL, CH_1_. Then, the ripple becomes the baseband offset, and the offset is integrated by the following Miller integrator. The integrated offset is modulated by the output chopper, CH_2_, and is negatively fed back to the input stage; thus, the ripple is reduced.

#### 2.2.3. Capacitive Impedance Boosting Loop (CIBL)

**Figure 11:**
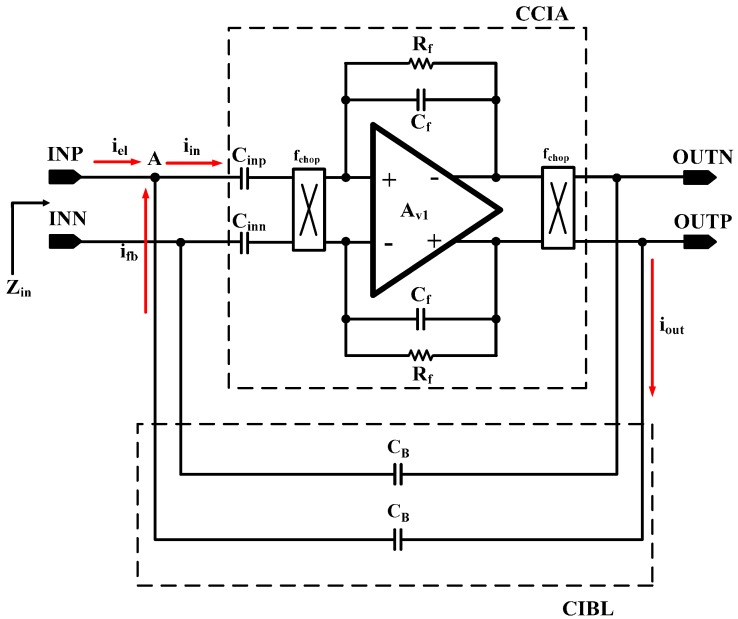
Schematic of the CIBL.

The schematic of the proposed capacitive input impedance boosting loop (CIBL) is shown in Figure 11. The proposed CIBL increases the input impedance and enhances the CMRR without additional power consumption. The pair of the capacitor connected at the CCIA input and output node forms the positive feedback loop.

The stability of the positive feedback is achieved by a slightly lower capacitor value at the positive feedback path than the feedback capacitor at the CCIA. The current from the electrode, *i_el_*, can be expressed as Equation (3), using the input current, *i_in_*, and the feedback current, *i_fb_*. The *i_fb_* is expressed as Equation (4). Thus, the input impedance, *Z_in_* can be expressed as Equation (5). As expressed in Equation (5), the current drawn from the electrode, *i_el_*, can be reduced by adding *C_B_* in the positive feedback path.

(3) $$i_{el}=i_{in}-i_{fb}$$

(4) $$i_{fb}=s\cdot C_{f}(V_{outp}-V_{inp})$$

(5) $$z_{in}=\frac{\left(V_{outp}-V_{outn}\right)}{i_{el}}=\frac{\left(V_{outp}-V_{outn}\right)}{i_{in}-s\cdot C_{B}(V_{outp}-V_{outn})}$$

## 3. Experimental Results

**Figure 12:**
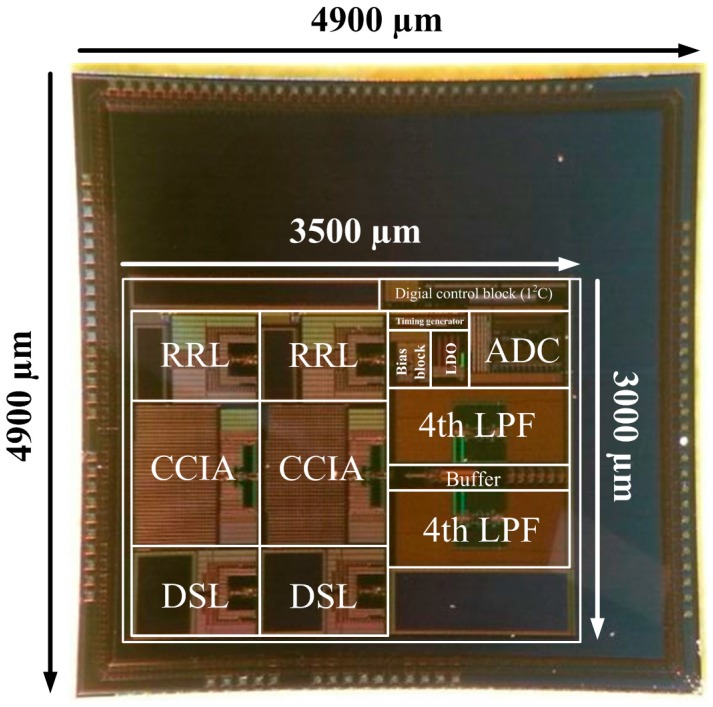
Microphotograph of the biopotential AFE IC.

Figure 12 shows a die photograph of the biopotential acquisition AFE IC. The IC is fabricated using a 1P6M 0.18 µm process. The core chip size of the biopotential acquisition AFE without I/O pads is 10.5 mm^2^. The AFE IC is fully integrated and does not require external components.

**Figure 13:**
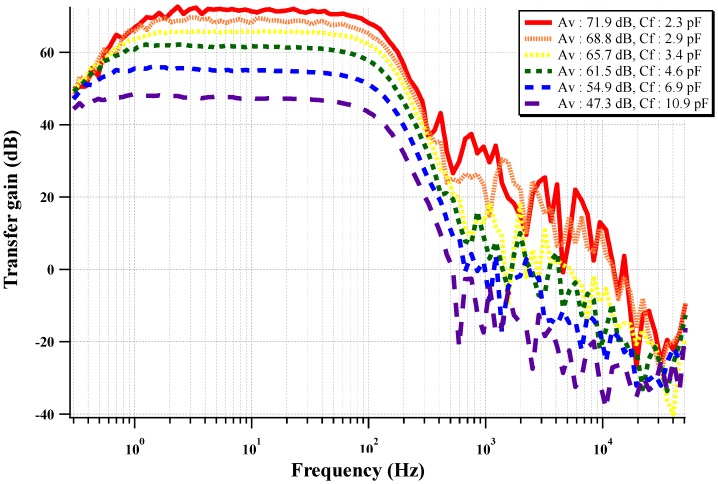
Measured frequency response of the two-stage CCIA with a varying feedback gain control capacitor.

The measured frequency response of the circuit is shown in Figure 13. The frequency response of the biopotential acquisition readout is a band-pass feature with a pass band of 1 to 100 Hz. The pass-band at the high frequency cut-off can be adjusted using a programmable resistor in the low-pass filter. The gain of the CCIA can be programmed with four-bit programmable feedback capacitors (Cf). The pass-band gain is programmable from 47.3 dB to 71.9 dB with four-bit resolution.

**Figure 14:**
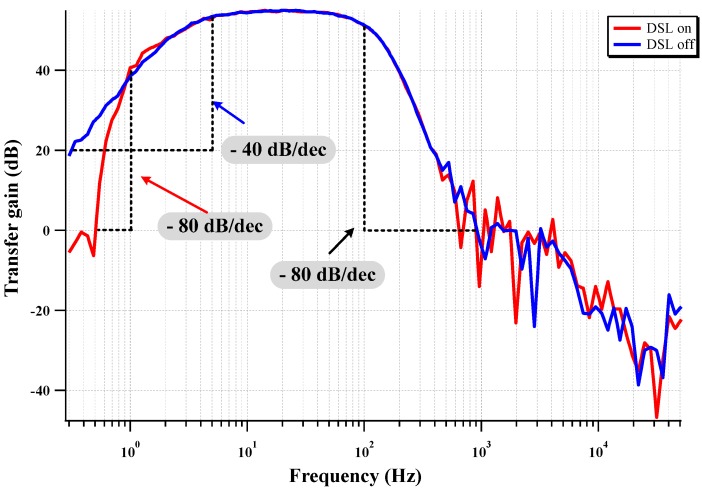
Measured frequency response of the DSL activation.

Figure 14 shows the frequency response when the DSL is activated. When the DSL at each CCIA is activated, the fourth-order HPF with 80 dB/dec slope is observed. The fourth-order LPF effectively attenuates the out-band components with a −80 dB/dec slope.

**Figure 15:**
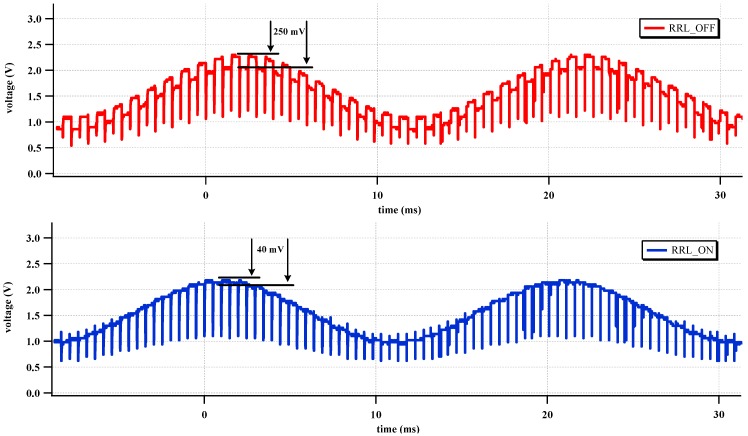
Measured time domain output with the RRL.

Figure 15 shows the effect of the RRL. The sinusoidal 1 mV signal at 50 Hz is used for the input source. The gain setting of the two-stage CCIA is 67 dB. The output ripple signal is decreased 84% by activating the RRL. The RRL operates as a notch filter at the chopper frequency and relaxes the requirements of the LPF in the output stage.

**Figure 16:**
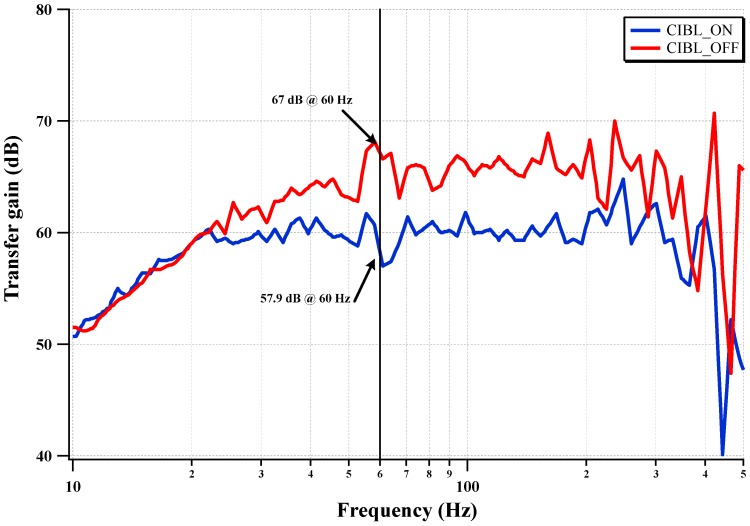
Measured CMRR with the CIBL.

The measured CMRR of the IC with CIBL activation is shown in Figure 16. The CIBL activation enhances the CMRR, even under electrode mismatch conditions, by boosting the input impedance. The two differential input electrodes are modeled using a 47 kΩ resistor and a 47 nanofarads capacitor in a parallel connection. The worst mismatch condition is assumed to be one electrode directly connected to the input terminal and the other electrode connected with the electrode model of a 47 kΩ resistor in parallel with a 47 nanofarads capacitor. The CMRR is improved from 57.9 dB to 67 dB (9.1 dB improvement) by activating the CIBL. The CMRR at 60 Hz is increased from 57.9 dB to 67 dB under the input electrode mismatch condition.

**Figure 17:**
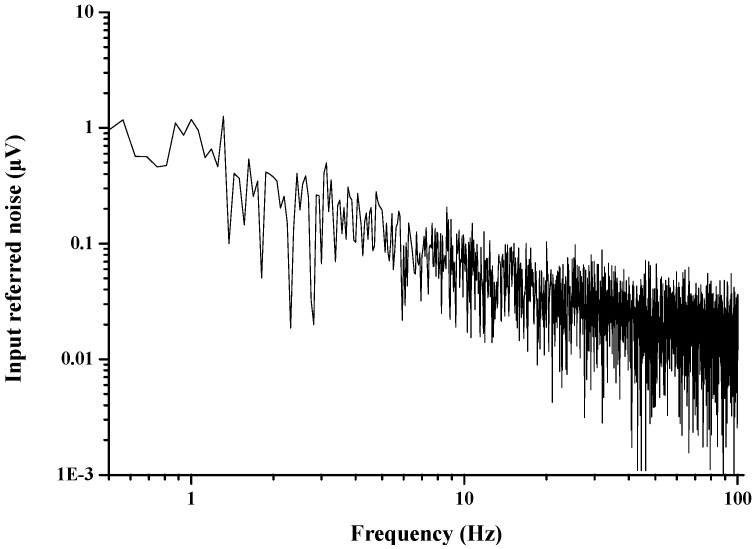
Measured IRN in band-width from 1 Hz to 100 Hz.

The measured IRN in the band-width from 1 Hz to 100 Hz is shown in Figure 17. The integrated IRN in the band-width from 1 Hz to 100 Hz is 0.94 μV_RMS_, when the DSL, RRL, and CIBL are activated.

**Figure 18:**
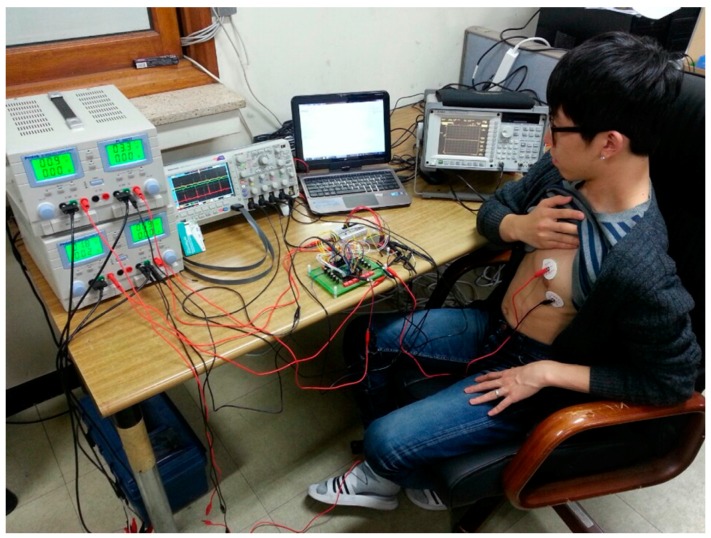
ECG signal measurement environment.

**Figure 19:**
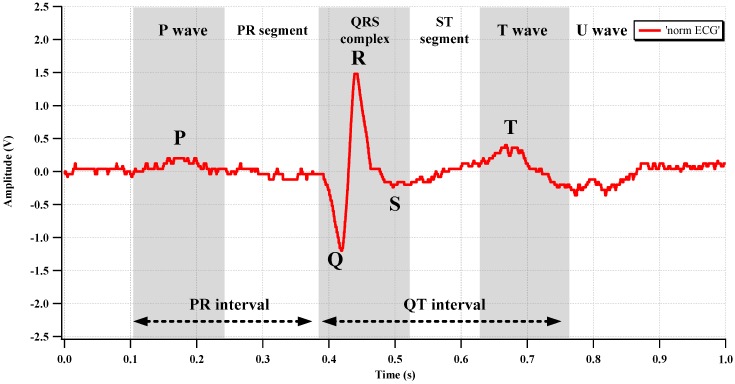
Measured ECG waveform.

To evaluate the biopotential recording capability of the fabricated IC, an ECG signal was acquired from the human body, as shown in Figure 18. Two electrodes, which are connected to the CCIA input port, were attached to the left breast near the heart. The AFE gain is set to 67.1 dB. The differential output of the recorded ECG is shown in Figure 19. The typical P-Q-R-S-T regions are clearly distinguishable.

**Figure 20:**
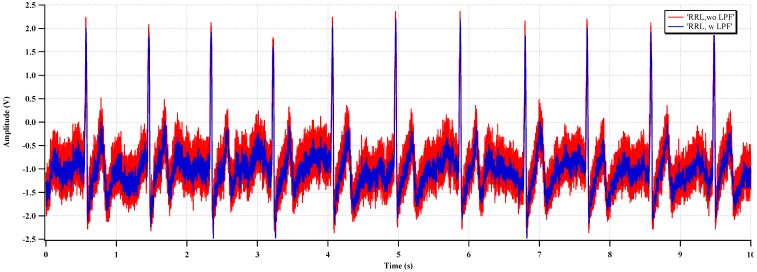
Measured ECG with RRL activation.

**Figure 21:**
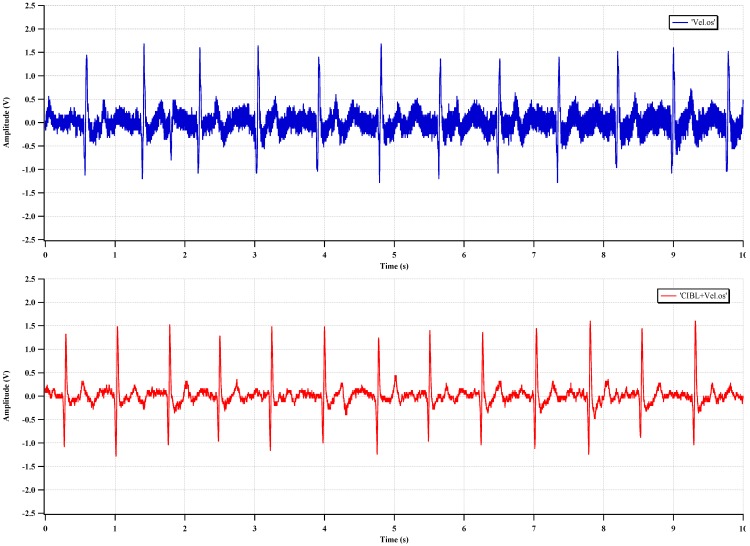
Measured ECG with CIBL activation.

The measured ECG waveforms with RRL and CIBL are shown in Figure 20 and Figure 21, respectively. In Figure 20, the high-frequency noise (ripple) is reduced, and a clear ECG waveform can be acquired, even without the LPF. In Figure 21, the common-mode noise, mainly 60-Hz interference, is reduced, and a clearer waveform is achieved by activating the CIBL.

**Figure 22:**
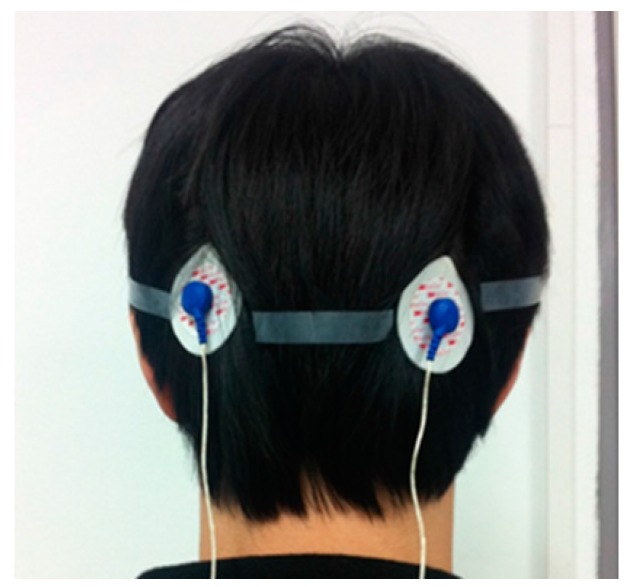
EEG signal measurement environment.

**Figure 23:**
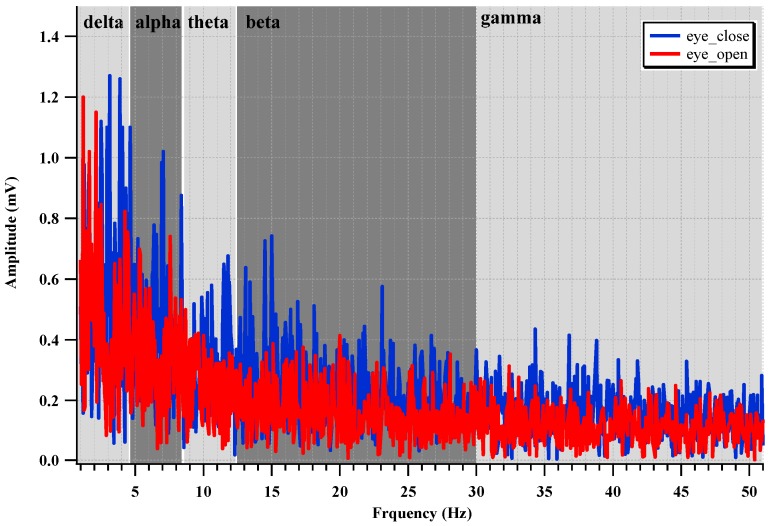
Measured EEG spectrum.

To evaluate the EEG recording performance of the fabricated IC, two electrodes are attached to the backside of the head, which is near the visual cortex, as shown in Figure 22. The alpha suppression phenomenon, which means that the alpha wave is suppressed when the eyes are opened, is measured. The measured EEG spectrum is shown in Figure 23. The average power when the eyes were open decreases by 61.2% compared with that when the eyes were closed.

## 4. Conclusions

The biopotential AFE IC with DSL, RRL, and CIBL is presented. The performance comparisons to previous research are summarized in Table 1 [5,6,7,8,9,10]. To compare the noise and power performance to previous researches, the noise efficiency factor (NEF) is used [11], as expressed in Equation (6):

(6) $$NEF=V_{rms,in}\sqrt{\frac{2I_{total}}{\pi \cdot U_{t}\cdot 4kT\cdot BW}}$$

where *V_rms,in_* is the input-referred RMS noise, *I_total_* is the total supply current, *U_t_* is the thermal voltage *kT/q*, and *BW* is the band-width of amplifier. The IC includes the two-stage chopper-stabilized CCIA to achieve low input referred noise. The CIBL is designed to enhance the input impedance and the CMRR. The DSL is designed to reduce the external offset and to implement additional HPF. The RRL is designed to reduce the chopper induced ripple. The IC is fabricated using a 0.18 µm 1P6M CMOS process. The core chip size of the IC without I/O pads is 10.5 mm^2^. The IC is fully integrated, and can be operated as a stand-alone biopotential measurement system with robust signal acquisition capability.

**Table 1:** Performance comparisons.

	This Work	Reference No.					
		[5]	[6]	[7]	[8]	[9]	[10]
Process (μm)	0.18	65n	0.5	0.35	0.18	0.8	0.5
VDD (V)	3.3	1	3	1.5	0.4	1.8	3
Current (μA)	3.8	1.8	20	0.18	0.226	1.2	485
Ripple reduction	Yes	Yes	No	No	No	No	No
DC servo loop	Yes	Yes	Yes (external capacitor)	No	No	No	No
Input impedance boosting	Yes	Yes	No	No	No	No	No
Passband (Hz)	0.5–100 (programmable)	/	0.5–150	20–280	0.5–100	0.5–250	0.3–150
Gain (dB)	71.9 (programmable)	40	60	40	40–70	45.5	80
CMRR (dB)	102 (wo/mismatch) 67 (w/mismatch)	134	110	74	120	100	110
Noise (μV) (100 Hz BW)	0.94	0.6	0.574	2.3	0.88	0.93	0.73
NEF	7.17	3.3	9.2	/	4.7	4.9	59
